# Unique Mutational Spectrum of the *GJB2* Gene and Its Pathogenic Contribution to Deafness in Tuvinians (Southern Siberia, Russia): A High Prevalence of Rare Variant c.516G>C (p.Trp172Cys)

**DOI:** 10.3390/genes10060429

**Published:** 2019-06-05

**Authors:** Olga L. Posukh, Marina V. Zytsar, Marita S. Bady-Khoo, Valeria Yu. Danilchenko, Ekaterina A. Maslova, Nikolay A. Barashkov, Alexander A. Bondar, Igor V. Morozov, Vladimir N. Maximov, Michael I. Voevoda

**Affiliations:** 1 Federal Research Center Institute of Cytology and Genetics, Siberian Branch of the Russian Academy of Sciences, 630090 Novosibirsk, Russia; zytzar@bionet.nsc.ru (M.V.Z.); danilchenko_valeri@mail.ru (V.Y.D.); maslova@bionet.nsc.ru (E.A.M.); medik11@mail.ru (V.N.M.); voevoda@bionet.nsc.ru (M.I.V.); 2 Novosibirsk State University, 630090 Novosibirsk, Russia; 3 Research Institute of Medical-Social Problems and Management of the Republic of Tyva, 667000 Kyzyl, Russia; marita.badyhoo@mail.ru; 4 Perinatal Center of the Republic of Tyva, 667000 Kyzyl, Russia; 5 Yakut Scientific Centre of Complex Medical Problems, 677019 Yakutsk, Russia; barashkov2004@mail.ru; 6 M.K. Ammosov North-Eastern Federal University, 677027 Yakutsk, Russia; 7 Institute of Chemical Biology and Fundamental Medicine, Siberian Branch of the Russian Academy of Sciences, 630090 Novosibirsk, Russia; alex.bondar@mail.ru (A.A.B.); Mor@niboch.nsc.ru (I.V.M.)

**Keywords:** hearing loss, nonsyndromic autosomal recessive deafness 1A (DFNB1A), *GJB2*, Tuvinians, Southern Siberia, Russia

## Abstract

Mutations in the *GJB2* gene are the main cause for nonsyndromic autosomal recessive deafness 1A (DFNB1A) in many populations. *GJB2* mutational spectrum and pathogenic contribution are widely varying in different populations. Significant efforts have been made worldwide to define DFNB1A molecular epidemiology, but this issue still remains open for some populations. The main aim of study is to estimate the DFNB1A prevalence and *GJB2* mutational spectrum in Tuvinians—an indigenous population of the Tyva Republic (Southern Siberia, Russia). Sanger sequencing was applied to analysis of coding (exon 2) and non-coding regions of *GJB2* in a cohort of Tuvinian patients with hearing impairments (*n* = 220) and ethnically matched controls (*n* = 157). Diagnosis of DFNB1A was established for 22.3% patients (28.8% of familial vs 18.6% of sporadic cases). Our results support that patients with monoallelic *GJB2* mutations (8.2%) are coincidental carriers. Recessive mutations p.Trp172Cys, c.-23+1G>A, c.235delC, c.299_300delAT, p.Val37Ile and several benign variants were found in examined patients. A striking finding was a high prevalence of rare variant p.Trp172Cys (c.516G>C) in Tuvinians accounting for 62.9% of all mutant *GJB2* alleles and a carrier frequency of 3.8% in controls. All obtained data provide important targeted information for genetic counseling of affected Tuvinian families and enrich current information on variability of *GJB2* worldwide.

## 1. Introduction

Hearing loss (HL) is one of the most common sensory disorders, affecting one in 500–1000 newborns, and approximately half of congenital HL cases have a genetic etiology [1]. The genetic causes for HL are extremely heterogeneous: over 400 syndromes are associated with HL [2] and over 100 different genes are causally implicated in nonsyndromic HL [3]. Despite the extraordinary genetic heterogeneity of nonsyndromic HL, homozygous or compound heterozygous mutations in the *GJB2* gene (gap junction protein, β-2, OMIM 121011, 13q12.11) encoding transmembrane protein connexin 26 (Cx26) are the most common cause of nonsyndromic autosomal recessive deafness 1A (DFNB1A, OMIM 220290) in many populations [4]. Six connexin 26 molecules associate to form transmembrane hexameric hemichannels (connexons) which dock with hemichannels of adjacent cells forming gap junctions that are essential for the transport of ions and other low-molecular-weight components between cells. Cx26 plays a crucial role in potassium homeostasis in the inner ear [5]. High prevalence of the *GJB2*-associated HL was demonstrated in most populations [1,6,7] making *GJB2* gene testing essential for the establishment of genetic diagnosis of HL.

Over 300 deafness-associated variations in *GJB2* have been reported in the Human Gene Mutation Database (http://www.hgmd.cf.ac.uk) [8]. Many of them have very specific ethno-geographic prevalence patterns [7,9] being attributed to a founder effect for certain ethnic groups. The variant c.35delG (p.Gly12Valfs*2) is prevalent in deaf patients of Caucasian origin [10,11]; c.235delC (p.Leu79Cysfs*3) is common in East and Southeast Asians [12,13,14]; c.167delT (p.Leu56Argfs*26) is more specific for Ashkenazi Jews [15]; c.427C>T (p.Arg143Trp) was found with high frequency in population of Ghana (West Africa) [16]; c.71G>A (p.Trp24*) is widely distributed in Indians and European Gypsies [17,18,19]; c.109G>A (p.Val37Ile) prevails in populations of Southeast Asia [20]; splice donor variant c.-23+1G>A (originally named IVS1+1G>A) is widespread among Yakuts (Eastern Siberia, Russia) [21]; c.131G>A (p.Trp44*) was found with high frequency among descendants of the ancestral Mayan population in Guatemala [22].

Despite significant efforts made worldwide to define the molecular epidemiology of *GJB2* pathogenic variants, this issue still remains open for some populations. Establishing a genetic diagnosis of HL is of great importance for clinical evaluation of deaf patients and for estimating recurrence risks for their families.

The aim of this work is to evaluate previously unknown contribution of the *GJB2* mutations to HL in Tuvinians, which is the indigenous population of the Tyva Republic. The Tyva Republic (Tuva), the federal subject of the Russian Federation, is located in Southern Siberia, in the central part of the Asian continent, bordered by Mongolia in the south and the east. The Tyva Republic is divided into 17 administrative districts and includes five small towns, with the capital in the town of Kyzyl. The total population of Tuva (307,930 according to 2010 Russian Federation Census) is mainly (82%) composed of the Tuvinians (Tuvans) who descended from ancient Turkic-speaking Central Asian tribes and Mongolian-speaking groups assimilated by them [23]. Besides the Tyva Republic, relatively small groups of Tuvinians live in Mongolia and in Northwest China [24].

In our previous epidemiological study, we created a database (including information about sex, age, ethnicity, place of residence, audiological data, and age of HL onset) for approximately 1400 individuals with various hearing impairments living in the Tyva Republic [25]. The average rate of different HL cases in the Tyva Republic was estimated as 45.5 ± 1.21 per 10,000 residents or 1:220, ranging from 25.5 ± 4.44 (1:392) to 117.9 ± 12.09 (1:85) in different regions of the Tyva Republic [25]. From the total group of registered patients, based on medical history, pedigree data and after exclusion of HL cases probably due to environmental factors, we have identified a group of patients with congenital or early onset sensorineural severe-profound HL (approximately 540 individuals) likely to have genetic HL. In this study, the evaluation of the spectrum and frequency of the *GJB2* gene variants was performed in a cohort of Tuvinians mainly recruited from this group of patients.

## 2. Materials and Methods 

### 2.1. Patients

The cohort of patients with hearing impairment (109 females and 111 males from 1 year to 70 years old at the time of investigation) includes 220 Tuvinians from 184 unrelated families living in the Tyva Republic (Southern Siberia, Russia). In total, 76 patients were urban residents (mainly from town Kyzyl) and 144 were rural residents from numerous small settlements located in different areas of the Tyva Republic. The genomic DNA samples of patients were collected from 2010 to 2018. Thorough analysis of pedigrees and family histories of patients was performed. The examined cohort of patients included 140 single/sporadic (the only affected individual in family) and 80 familial (two or more affected family members) HL cases. Hearing status of parents was ascertained for 210 patients: 186 (88.6%) patients have both normal hearing parents (mating N x N), 9 patients (4.3%) have one deaf and another normal hearing parents (mating D x N), 15 (7.1%) patients were descendants of assortative matings (both deaf parents, mating D x D), 10 of which could be considered, according to [26], as non-complementary matings (noncomp. mating D x D) that is, the families with both deaf parents and all deaf siblings presumably having the same genetic cause of HL.

Hearing status of affected individuals was evaluated by otoscopic and pure-tone audiometry examinations that patients underwent at different times in the specialized audiological services located in town Kyzyl. Severity of hearing loss was defined as mild (25–40 dB), moderate (41–70 dB), severe (71–90 dB) or profound (above 90 dB). The majority of examined patients (210 subjects) suffered from congenital or early onset bilateral severe-to-profound HL, nine patients have moderate HL, and one patient – unilateral severe HL. Other concomitant information was collected from local unspecialized medical services and by direct interview with the patients and their relatives.

### 2.2. Control Sample

The ethnically matched control sample comprises 157 unrelated Tuvinians from different regions of the Tyva Republic (84 females and 73 males, aged from 10 to 73 years old). None of them were registered by the audiological services and had no complaints of hearing impairments.

### 2.3. Ethics Statement

Written informed consent was obtained from all individuals or their legal guardians before they participated in the study. The study was conducted in accordance with the Declaration of Helsinki, and the protocol was approved by the Bioethics Commission at the Institute of Cytology and Genetics SB RAS, Novosibirsk, Russia (Protocol No. 9, 24 April 2012).

### 2.4. Mutation Analysis of the GJB2 Gene

Genomic DNA was isolated from the buffy coat fraction of blood by a standard phenol-chloroform extraction method. The *GJB2* coding region encompassing exon 2 and the non-coding *GJB2* region (exon 1 with flanking regions) were amplified in all patients and controls. Primer pairs designed to amplify corresponding PCR products and also used for Sanger sequencing are presented in Table 1. The PCR products were purified by sorption on Agencourt Ampure XP (Beckman Coulter, Indianapolis, IN, USA) and subjected to Sanger sequencing using a BigDye Terminator V.3.1 Cycle Sequencing Kit (Applied Biosystems, Waltham, MA, USA) with subsequent unincorporated dye removal by the Sephadex G-50 gel filtration (GE Healthcare, Chicago, IL, USA). The Sanger products were analyzed on an ABI 3130XL Genetic Analyzer (Applied Biosystems) in the SB RAS Genomics Core Facility (Institute of Chemical Biology and Fundamental Medicine SB RAS, Novosibirsk, Russia). The DNA sequence variations were identified by comparison with the *GJB2* gene reference sequence NG_008358.1 (https://www.ncbi.nlm.nih.gov/nuccore/NG_008358.1). Screening of common large deletion GJB6-D13S1830 (~309 kb) was performed according to [27,28] only for the group of patients with monoallelic *GJB2* recessive mutations. In this group of patients, we also sequenced a region of about 0.7 kb immediately upstream of *GJB2* exon 1 which includes the *GJB2* basal promoter using primers PF1, PF2, and PR1 as previously described [29] (Table 1).

### 2.5. Verification of Cis-Configuration of GJB2 Variants c.79G>A (p.Val27Ile) and c.341A>G (p.Glu114Gly)

*GJB2* variants c.79G>A (p.Val27Ile, rs2274084) and c.341A>G (p.Glu114Gly, rs2274083) are located 260 bp from each other in the *GJB2* coding sequence. The phase (*cis* or *trans*) of these variants was assessed in all individuals who were heterozygous for both of these variants (deaf patients, their relatives and individuals from the control sample) by either pedigree analysis or by molecular cloning and Sanger sequencing of the syntenic variants (Figure S1). Molecular cloning was performed for 27 individuals heterozygous for both of these variants by use of the CloneJET™ PCR Cloning Kit (Thermo Fisher Scientific, Waltham, MA, USA) according to the manufacturer’s protocol. The fragment of the *GJB2* coding region (837 bp) including both variants c.79G>A and c.341A>G was PCR-amplified from genomic DNA using Phusion Hot Start II polymerase (Thermo Fisher Scientific) and primers 835-F and 835-R (Table 1) and then cloned into the pJET1.2/blunt vector followed by transformation in competent *E.coli* strain Mach-1 (Invitrogen, Carlsbad, CA, USA). Presence/absence of variants c.79G>A and c.341A>G in examined positive clones was verified by Sanger sequencing with the same primers (Figure S1).

### 2.6. Bioinformatics Prediction Tools

The functional effects of the c.516G>C (p.Trp172Cys) variant were predicted using PolyPhen-2 (http://genetics.bwh.harvard.edu/pph2) [31], PROVEAN (http://provean.jcvi.org) [32], PANTHER (http://www.pantherdb.org) [33], MutationTaster (http://www.mutationtaster.org/) [34], and FATHMM (http://fathmm.biocompute.org.uk/index.html) [35].

### 2.7. 3D Cx26 Molecule Structure Modelling

The three-dimensional (3D) molecule structure of the wild type and the p.Trp172Cys-mutant connexin 26 (Cx26-WT and Cx26-p.Trp172Cys, respectively) was visualized by DeepView/Swiss-PdbViewer v.4.1.0 (http://www.expasy.org/spdbv/) [36]. The protein template 2ZW3.1 was found by the SWISS-MODEL (http://swissmodel.expasy.org/) [36].

### 2.8. Statistical Methods

Two-tailed Fisher’s exact test with significance level of *p* < 0.05 was applied to compare allele frequencies between patients and controls. To avoid probable bias due to the presence of related individuals in the total cohort of patients, only unrelated patients (184 out of 220 selected by pedigree analysis) were used for allele frequency calculation.

## 3. Results

### 3.1. GJB2 Genotypes Observed in Patients and Control Sample

Five pathogenic recessive *GJB2* variants: c.516G>C (p.Trp172Cys), c.-23+1G>A, c.235delC (p.Leu79Cysfs*3), c.299_300delAT (p.His100Argfs*14), c.109G>A (p.Val37Ile), and also several known benign *GJB2* variants were found in examined patients. The *GJB2* genotypes due to the combination of *GJB2* gene sequence variations observed in patients and in the control sample are presented in Table 2. 

Twenty-two different *GJB2* genotypes found in patients can be subdivided into four groups: (1) seven genotypes with biallelic (homozygous or compound heterozygous) recessive pathogenic *GJB2* variants (22.3%, 49 out of 220 patients); (2) six genotypes with single recessive pathogenic *GJB2* variants in compound with a benign variant or wild type allele (8.2%, 18 out of 220 patients); (3) eight genotypes with only benign *GJB2* variants (24.5%, 54 out of 220 patients); (4) wild genotype (without any changes in the *GJB2* gene sequence) (45.0%, 99 out of 220 patients).

Twelve different *GJB2* genotypes (including wild genotype) were found in the control sample: five genotypes included a single recessive pathogenic *GJB2* variant in compound with a benign variant or wild type allele (9.6%, 15 out of 157 individuals); six genotypes—only benign *GJB2* variants (33.7%, 53 out of 157 individuals); wild genotype (56.7%, 89 out of 157 individuals) (Table 2).

The contribution of the *GJB2* pathogenic variants in HL of patients defined as the proportion of patients from the first group of *GJB2* genotypes among all examined Tuvinian patients was estimated as 22.3%. The c.516G>C (p.Trp172Cys) variant was the most frequent (62.9%) out of all *GJB2* pathogenic variants detected in patients followed by c.-23+1G>A (27.6%), c.235delC (5.2%), c.299_300delAT (2.6%), and c.109G>A (p.Val37Ile) (1.7%). Total carrier frequency of the *GJB2* pathogenic variants in Tuvinian control sample was estimated as 9.6% (p.Trp172Cys – 3.8%, c.-23+1G>A – 3.8%, and p.Val37Ile – 1.9%).

### 3.2. GJB2 Sequence Variations in Patients and Control Sample

The c.516G>C (p.Trp172Cys) variant makes a significant contribution to deafness in Tuvinian patients since 38 (from 27 unrelated families) out of 220 examined patients are either homozygotes or compound heterozygotes for p.Trp172Cys, and also 10 patients (from seven unrelated families) have only a single allele with p.Trp172Cys (Table 2). The p.Trp172Cys variant (in compound with c.235delC) was initially found in our previous study in one deaf patient belonging to Altaians—indigenous Turkic-speaking people of the Altai Republic neighboring the Tyva Republic (Southern Siberia, Russia) [37]. Later, Tekin et al. (2010) detected p.Trp172Cys in compound with mutation c.299_300delAT in one deaf patient from Mongolia [38]. 

**Figure 1:**
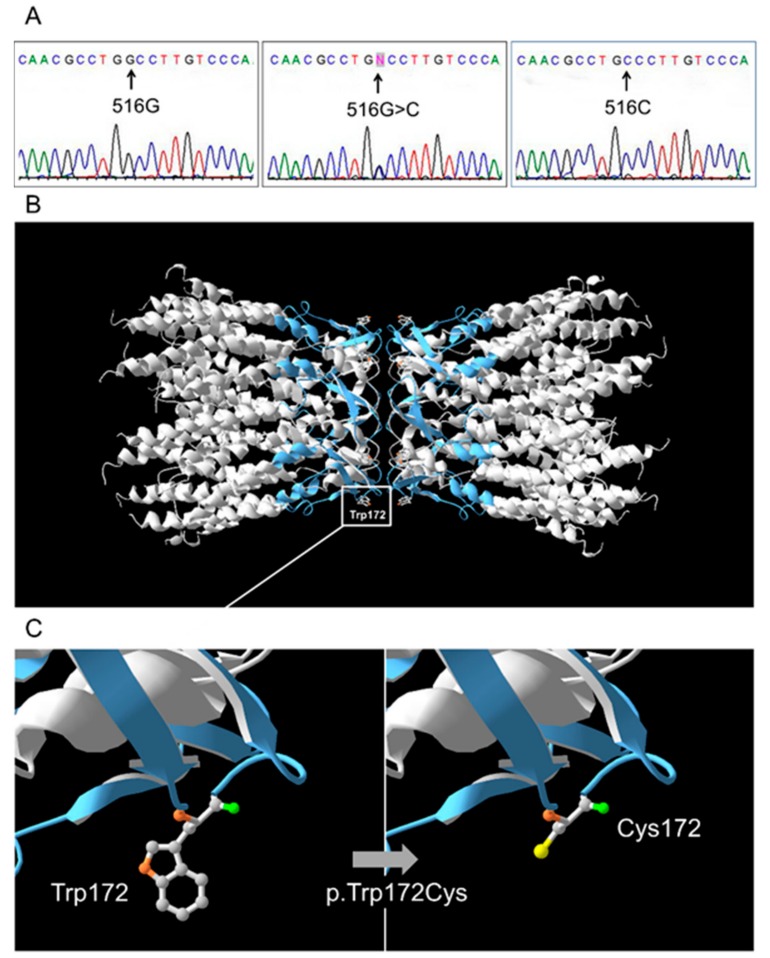
(**A**) Identification of the c.516G>C (p.Trp172Cys) mutation by Sanger sequencing. (**B**) The 3D structure of normal protein connexin 26 (Cx26-WT). Two adjacent Cx26-WT hemichannel subunits with designation of Trp (tryptophan) on position 172. The extracellular loop of Cx26 (E2, 155-192 amino acid residues) where variant p.Trp172Cys located is marked by blue. (**C**) Close-up view of Cx26-WT (Trp172) and mutant Cx26-p.Trp172Cys (Cys172) connexin 26.

The c.516G>C substitution leads to a replacement of aromatic non-polar tryptophan by a small polar cysteine at amino acid position 172 in the second extracellular loop (E2, 155-192 amino acid residues) of the connexin 26 protein (Cx26) (Figure 1). The loop E2 is known to be essential for Cx26-hemichannel subunits connection [39]. Multiple in silico programs (scores: PolyPhen2—‘probably/possibly damaging’, PROVEAN—‘deleterious’, PANTHER—‘probably damaging’, MutationTaster—‘disease causing’, FATHMM—‘damaging’) predict a deleterious effect of p.Trp172Cys. For now, variant p.Trp172Cys is only presented in the Human Gene Mutation Database (HGMD: http://www.hgmd.cf.ac.uk/ac/) [8] with reference to our previous work [37].

**Figure 2:**
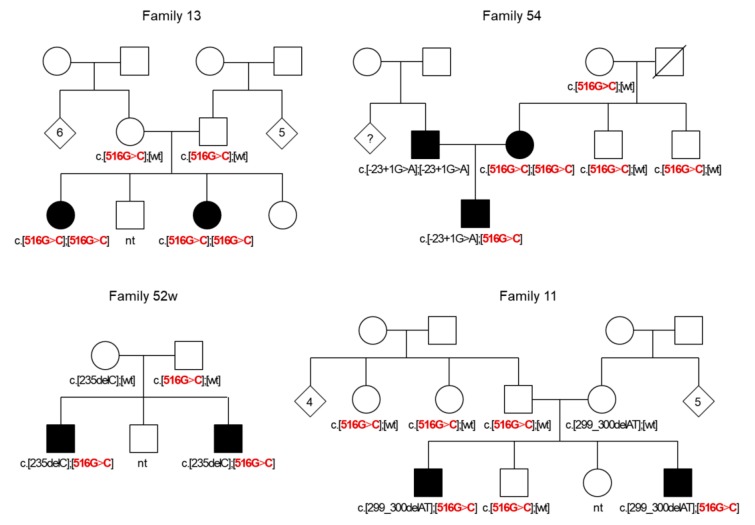
The pedigrees of Tuvinian families demonstrating the segregation of variant c.516G>C (p.Trp172Cys) in a homozygous state or in compound with other recessive *GJB2* mutations (c.235delC, c.-23+1G>A, and c.299_300delAT) with HL in affected family members. Deaf individuals are shown by black symbols. The variant c.516G>C (p.Trp172Cys) is shown by red.

All 38 examined patients having p.Trp172Cys (biallelic or in compound with known pathogenic *GJB2* variants c.-23+1G>A, c.235delC, or c.299_300delAT) were affected by nonsyndromic congenital or early onset HL. To investigate the segregation of p.Trp172Cys with HL, we have screened this variant in all available relatives of these patients and revealed a distinct segregation of homozygosity or compound heterozygosity for p.Trp172Cys with HL in affected Tuvinian families (Figure 2 and Figure S2). Moreover, the frequency of p.Trp172Cys calculated in unrelated patients (0.1386, 51/368 chromosomes) was significantly higher compared with the control sample (0.019, 6/314 chromosomes) (*p* < 10^−8^) (Table 3).

The splice donor site variant c.-23+1G>A (rs80338940) located in the *GJB2* non-coding region (intron 1) is the second common pathogenic *GJB2* mutation (after p.Trp172Cys) in Tuvinian patients and controls. The c.-23+1G>A is apparently not a rare *GJB2* mutation since it was found among deaf patients of different origins around the world [9,21,30,38,40,41,42,43,44] although not all laboratories are screening for this particular mutation since it is located outside the *GJB2* coding region. The extensive accumulation of c.-23+1G>A was reported in Yakuts, indigenous Turkic-speaking people living in the subarctic region of Russia (the Sakha Republic/Yakutia). Extremely high prevalence of c.-23+1G>A could be explained by a founder effect and a probable selective advantage for the c.-23+1G>A heterozygotes in severe subarctic climate [21,43]. The c.-23+1G>A was reported as the most common *GJB2* mutation in deaf Mongolian patients from Mongolia [38,44].

The *GJB2* frameshift mutations c.235delC (rs80338943) and c.299_300delAT (rs111033204) found in Tuvinian patients are well known deleterious *GJB2* variants associated with deafness. In many extensive studies, c.235delC and c.299_300delAT have been confirmed to be the most common *GJB2* mutations in the East Asian (Chinese, Taiwanese, Japanese, Korean) populations [7,9,12,13,14,45,46] as well as in Mongolian patients living in Mongolia and in the Inner Mongolia Autonomous Region of China [38,44,47].

Mutation c.109G>A (p.Val37Ile, rs72474224) which is predominant in Southeast Asian populations [7,9,20] was rare in Tuvinian patients—only two of them had compound heterozygous and heterozygous p.Val37Ile (Table 2).

The *GJB2* variants c.79G>A (p.Val27Ile, rs2274084) and c.341A>G (p.Glu114Gly, rs2274083) found in Tuvinian patients and control individuals (Table 2) are known to be common in many Asian populations. The p.Val27Ile can be detected as a single variation as well as together with p.Glu114Gly, while p.Glu114Gly is very rarely found alone. These variants are mainly considered as benign polymorphisms due to their presence in deaf patients and controls; however, some studies have not excluded a possible association of these variants combination (in particular, related to their configuration - *cis* or *trans*) with HL [38,48,49,50,51]. In vitro functional analyses were performed to clarify the possible role of p.Val27Ile and p.Glu114Gly in HL [38,49,52]. Choung et al. (2002) investigated HeLa cells expressing single variant p.Glu114Gly and revealed their gap junction functioning similar to wild type cells [52]. Tekin et al. (2010) showed that p.Val27Ile + p.Glu114Gly can significantly impair the function of the gap junctions but there were no differences in the p.Val27Ile + p.Glu114Gly haplotype frequencies between patients and control group [38]. Choi et al. (2011) showed reduced activity of the p.Glu114Gly and the p.Val27Ile + p.Glu114Gly channels and suggested that only the p.Glu114Gly homozygote or compound heterozygote for p.Glu114Gly and other *GJB2* pathogenic variant can cause HL [49]. Thus, the pathogenicity of these variants remains controversial and is widely discussed in literature.

In our study, 17 Tuvinian patients and 15 control individuals were heterozygous for both p.Val27Ile and p.Glu114Gly and none of the examined individuals had p.Glu114Gly separately from p.Val27Ile (Table 2). We investigated the phase (*cis* or *trans*) of p.Val27Ile and p.Glu114Gly in all individuals heterozygous for these variants by analysis of pedigrees of patients (if relevant data were available) or by sequencing of cloned fragments spanning the variants (Figure S1). In this way, we proved their *cis*-configuration in all cases where p.Glu114Gly was found. Thus, we postulated the presence of allele p.[Val27Ile;Glu114Gly] in our examined samples and estimated its frequency in patients and controls as 0.0408 and 0.0478, respectively. Since the frequency of p.[Val27Ile;Glu114Gly] was slightly higher in controls than in patients (although the difference was not statistically significant, *p* = 0.3969), we believe that there is no association of allele p.[Val27Ile;Glu114Gly] with HL, at least in our sample of patients.

Other *GJB2* benign variants found in Tuvinians were c.571T>C (p.Phe191Leu, rs397516878, c.457G>A (p.Val153Ile, rs111033186), and c.608T>C (p.Ile203Thr, rs76838169). They are listed as rare polymorphisms preferentially detected in South Asian populations (dbSNP: https://www.ncbi.nlm.nih.gov/snp/).

### 3.3. Group of Patients with a Single GJB2 Pathogenic Variant

We found that 18 among all 220 examined patients (8.2%) are the carriers of a single recessive *GJB2* pathogenic variant: 10 patients have single c.516G>C (p.Trp172Cys), 6 patients - c.-23+1G>A, 1 patient - c.235delC, and 1 patient - c.109G>A (p.Val37Ile) (Table 2). We analyzed the pedigree and family data of patients with *GJB2* monoallelic mutations. These patients represented eleven sporadic and seven familial HL cases. Among familial cases, four patients were descendants of both deaf parents (mating D x D), two patients had normal hearing parents (mating N x N), and one patient was from mating D x N (Table S1 and Figure S3). 

A relatively large proportion of deaf individuals carrying only one pathogenic recessive *GJB2* variant has been reported in many previous studies [6,7,41,53,54,55,56,57,58,59,60,61]. Two main assumptions have been made to resolve this issue: (1) HL of such patients could be caused by an uncertain impact of the *GJB2* gene (the presence of yet undetected other pathogenic *GJB2* variants or variable penetrance of pathogenic *GJB2* variant due to any modulating factors); (2) these patients are only the coincidental carriers of one pathogenic *GJB2* allele and other factors (other genes or environmental impacts) cause their HL.

The coding region of the *GJB2* gene and the region where splice donor variant c.-23+1G>A is located were routinely analyzed in majority of studies while the involvement of other *GJB2* noncoding regions in HL has not been extensively investigated. The *GJB2* gene has a 128 bp basal promoter including a TATA-box (TTAAAA) at -19 to -24 and 2 GC-boxes (CCGCCC) at -76 to -81 and -88 to -93 proximal to the transcriptional start site [62]. 

Several studies have reported that some noncoding *GJB2* variants found in upstream and basal promoter regions, exon 1, and in splice acceptor site may contribute to HL [29,63,64,65,66]. In addition, large deletions which either eliminate a hypothesized *cis*-acting regulatory element located far upstream of *GJB2* or encompass the complete *GJB2* and *GJB6* genes have been reported [27,67,68,69,70,71].

We tried to clarify the cause of HL in Tuvinian patients with monoallelic *GJB2* mutations. To search additional (probably missed in routine *GJB2* analysis) alterations in the potentially regulatory region of *GJB2*, for 18 DNA samples of these patients we sequenced the 1009 bp region immediately upstream of *GJB2* exon 1, which includes the basal promoter according to [29]. We also reanalyzed the *GJB2* region where the acceptor splice site of the only *GJB2* intron is located [66]. 

All variations found in these *GJB2* regions are presented in Table S1. No known pathogenic variants, including c.-684-675del (previously named as −493del10 [63]), were found in patients with monoallelic *GJB2* mutations. We had also screened the most common deletion *GJB6*-D13S1830 [27] in *GJB2* heterozygous patients. None of these patients had this deletion.

Moreover, at the time of this study, our group began to test the *SLC26A4* gene (OMIM 605646, 7q22.3) as a potential candidate gene for HL in *GJB2*-negative patients including 18 *GJB2* heterozygous patients. Our preliminary results of *SLC26A4* testing suggest that HL of at least 4 out of 18 (22%) patients with monoallelic *GJB2* mutations was caused by homozygosity or compound heterozygosity for *SLC26A4* pathogenic variants (Table S1), and consequently these patients are definitely the coincidental carriers of one pathogenic *GJB2* allele. 

Assuming that HL in patients with monoallelic *GJB2* mutations is not associated with the *GJB2* gene, the frequency of *GJB2* mutations in a sample of non-DFNB1A patients (that is, patients that do not have biallelic *GJB2* mutations) should be the same as the frequency in control samples. To test this hypothesis, we compared the frequencies of *GJB2* pathogenic variants among non-DFNB1A patients and controls. To avoid probable bias due to the presence of related individuals in the cohort of Tuvinian patients, only unrelated patients selected by pedigree analysis (in total, 146 patients including 14 *GJB2* heterozygous patients and 132 patients without the *GJB2* mutations) were used for this calculation. Overall frequency of monoallelic *GJB2* mutations among this group of patients was calculated to be 0.0479. Among control samples, 15 individuals were heterozygous for one of any *GJB2* mutations (Table 2), and thus the total frequency of the *GJB2* mutations was estimated as 0.0478 (Table 3). As a result, there were no statistically significant differences in the frequency of monoallelic *GJB2* mutations among patients compared to the control sample.

## 4. Discussion

A high variability in the *GJB2* mutation spectrum and frequencies has been reported among different worldwide populations. Nevertheless, the contribution of the *GJB2* gene to HL (DFNB1A) remains unknown for certain ethnic groups in different regions of the world.

In this study, we present for the first time data on the spectrum and frequency of the *GJB2* gene variants in a large cohort of Tuvinian patients with HL (*n* = 220) and also in unrelated normal hearing Tuvinians (*n* = 157) living in the Tyva Republic (Southern Siberia, Russia). As the result of sequencing the *GJB2* coding (exon 2) and non-coding (exon 1) regions with flanking sequences, a genetic diagnosis “Nonsyndromic autosomal recessive deafness 1A (DFNB1A)” due to the presence of biallelic *GJB2* pathogenic variants was established for 22.3% (49 out of 220) patients. In total, 8.2% (18 out of 220) of patients were the carriers of a single recessive *GJB2* pathogenic variant, and 69.5% (153 out of 220) of patients were *GJB2*-negative (without *GJB2* pathogenic variants).

In total, 26 out of 49 DFNB1A-patients represent sporadic HL cases and 23–familial cases. Thus, the DFNB1A prevalence among all sporadic cases (*n* = 140) could be estimated as 18.6%, whereas the proportion of DFNB1A among familial cases (*n* = 80)—as 28.8%. A lower frequency of DFNB1A in sporadic cases compared with familial cases may partly be explained by the inclusion of potential nongenetic HL cases. Based on the pedigree data, we found that 20 out of 23 familial DFNB1A-patients were from 11 unrelated families with two and more deaf siblings representing typical autosomal recessive type of HL inheritance, and although not all affected individuals in these families were examined by us, untested siblings in these Tuvinian families obviously have the same type of HL (DFNB1A). Two familial DFNB1A-cases were represented by patients who were descendants from non-complementary assortative matings (noncomp. mating D x D) [26]; therefore, HL of their untested deaf parents and siblings is also highly likely due to the *GJB2* mutations. The last familial DFNB1A-patient was from mating D x N, representing a pseudo-dominant type of inheritance. Thus, based on pedigree data, we suggest that contribution of the *GJB2* mutations to HL in deaf Tuvinians may be more than 22.3% due to probable DFNB1A-cases in untested affected relatives of examined patients, making our results applicable in genetic testing and counseling for a larger number of deaf patients in the Tyva Republic.

A large number of deaf individuals with a single recessive *GJB2* pathogenic allele was detected in different studies, which complicates the molecular diagnosis for their HL [6,7,41,53,54,55,56,57,58,59,60,61,72]. These findings may partly be explained by the fact that only the *GJB2* coding region (exon 2) was tested in some older studies; therefore, potential pathogenic variations in the *GJB2* non-coding or regulatory regions could be missed.

We thoroughly analyzed a group of 18 Tuvinian patients with a single recessive *GJB2* pathogenic variant (8.2% out of all examined patients) and concluded that they are most likely the coincidental carriers of one pathogenic *GJB2* allele and the cause of their HL could be attributed to mutations in other genes associated with HL or environmental impacts. The absence of differences in the frequency of monoallelic *GJB2* mutations in patients and in the control sample may be additional evidence for this conclusion.

Ten *GJB2* sequence variations including five pathogenic (p.Trp172Cys, c.-23+1G>A, c.235delC, c.299_300delAT, and p.Val37Ile) and five benign (p.Val27Ile, p.Glu114Gly, p.Phe191Leu, p.Val153Ile, and p.Ile203Thr) variants were identified among 220 examined Tuvinian patients. 

A striking finding was the discovery of common *GJB2* variant c.516G>C (p.Trp172Cys) found with frequency 62.9% among all *GJB2* mutated alleles detected in deaf Tuvinian patients. The p.Trp172Cys carrier frequency in Tuvinian control group was estimated as 3.8%.

The c.516G>C substitution leads to a replacement of aromatic non-polar tryptophan by a small polar cysteine at conservative amino acid position 172 (p.Trp172Cys) in the second extracellular loop (E2, 155-192 amino acid residues) of protein Cx26 (Figure 1). Both extracellular loops (E1 and E2) of Cx26 molecules contain six conserved cysteines (C53, C60, C64 in E1 and C169, C174, C180 in E2) which form intramolecular disulfide bonds, playing an essential role in Cx26 hemichannel subunits docking [39]. To our knowledge, the only other substitution of tryptophan at position 172 to arginine (p.Trp172Arg) caused by substitution c.514T>A has been described in the study by Mani et al. (2009) where two deaf patients from India were found to be homozygous for p.Trp172Arg [64]. Mani et al. showed that Trp172Arg-mutated Cx26 exhibited normal plasma membrane localization but defective gap junction channel activity and the authors suggested that replacement of tryptophan 172, located in close proximity to at least two of three cysteine residues in E2, could result in defective docking of two opposing connexon hemichannels [64].

Other lines of evidences also support pathogenicity of c.516G>C (p.Trp172Cys). A distinct segregation of homozygosity or compound heterozygosity for p.Trp172Cys with HL was revealed in affected Tuvinian families, and the p.Trp172Cys frequency among Tuvinian patients (0.1386) was significantly higher than in Tuvinian controls (0.019) (*p* < 10^−8^). Multiple in silico tools (PolyPhen2, PROVEAN, PANTHER, MutationTaster, FATHMM) predict a deleterious effect of p.Trp172Cys. 

The p.Trp172Cys variant is yet unreported in the world human genome databases (dbSNP, Exome Variant Server, Exome Aggregation Consortium (ExAC), 1000 Genomes Project). Recently, we submitted c.516G>C (p.Trp172Cys) to ClinVar (http://www.ncbi.nlm.nih.gov/clinvar/) as a ‘likely pathogenic’ variant (ClinVar accession number SCV000852065) because p.Trp172Cys meets main genetic criteria [73] for such classification: (i) strong segregation of homozygosity for p.Trp172Cys with recessive HL in multiple affected subjects from unrelated families; (ii) significantly higher frequency of p.Trp172Cys in patients compared with ethnically matched controls; (iii) multiple computational predictions of deleterious effect of p.Trp172Cys; (iiii) extreme rarity of p.Trp172Cys.

Chronologically, the p.Trp172Cys variant was initially found in our previous study in one patient belonging to Altaians - indigenous Turkic-speaking people of the Altai Republic (Southern Siberia, Russia) neighboring the Tyva Republic [37] and later Tekin et al. (2010) detected p.Trp172Cys in one Mongolian deaf patient from Mongolia [38]. Our recent data obtained on extended sample of Altaian deaf patients [74] revealed slightly elevated prevalence of p.Trp172Cys in Altaians whereas recent studies did not detect p.Trp172Cys among deaf Mongolian patients from Mongolia [44] as well as in Mongolian patients living in China [47,75]. Therefore, we suggest that p.Trp172Cys is the *GJB2* mutation endemic to Tuvinians, indigenous Turkic-speaking people of the Tyva Republic, since apart from them p.Trp172Cys has only minor incidence in indigenous peoples living in the neighboring Altai Republic and Mongolia.

## 5. Conclusions

The data on spectrum and frequency of *GJB2* gene variants were obtained for the first time for a large cohort of 220 deaf Tuvinian patients from the Tyva Republic (Southern Siberia, Russia) and ethnically matched cohort of 157 subjects with normal hearing. The *GJB2* biallelic pathogenic variants were found in 22.3% patients. We suggest that patients with monoallelic *GJB2* mutations (8.2%) are coincidental carriers. High prevalence of rare pathogenic variant p.Trp172Cys was found in Tuvinians while other *GJB2* mutations are shared by nearby populations. All obtained data provide important targeted information for genetic counseling of affected Tuvinian families and enrich current information on variability of *GJB2* worldwide.

## Figures and Tables

**Table 1:** Primers for polymerase chain reaction (PCR)/Sanger sequencing.

*GJB2*	Primers	Reference
*GJB2* coding region (exon 2) and flanking sequences	835-F: 5’-TGCTTGCTTACCCAGACTCA-3’ 835-R: 5’-CCTCATCCCTCTCATGCTGT-3’	This study
	or	
	F3044: 5’-AGTGCCTTTCAGCTAACGA-3’ R4242: 5’-GTGGCATCTGGAGTTTCACC-3’	
Upstream region of exon 1 (which includes the basal promoter), exon 1, donor splice site, and flanking intronic region	Ex1-F: 5’-TCTTTTCCAGAGCAAACCGC-3’ Ex1-R: 5’-CTGGGCAATGCGTTAAACTGG-3’	[30]
	Ex1-792-F: 5’-GCGTTCGTTCGGATTGGT-3’ Ex1-2239-R: 5’-CGGAAACAGACCCTCGTGAAGT-3’	This study
	PF1: 5’- GGCTCAAAGGAACTAGGAGATCG-3’ PF2: 5’-CGTTCGTTCGGATTGGTGAG-3’PR1: 5’-CAGAAACGCCCGCTCCAGAA-3’	[29]

F: Forward; R: Reverse.

**Table 2:** The *GJB2* genotypes found in Tuvinian patients and control sample.

*GJB2* Genotypes *	Patients (*n* = 220)	Control Sample (*n* = 157)
***GJB2* genotypes with biallelic recessive pathogenic variants**		
c.[516G>C];[516G>C] p.[Trp172Cys];[Trp172Cys]	25	-
c.[-23+1G>A];[-23+1G>A] p.[splice donor variant];[splice donor variant]	9	-
c.[-23+1G>A];[516G>C] p.[splice donor variant];[Trp172Cys]	7	-
c.[235delC];[516G>C] p.[Leu79Cysfs*3];[Trp172Cys]	4	-
c.[299_300delAT];[516G>C] p.[His100Argfs*14];[Trp172Cys]	2	-
c.[-23+1G>A];[299_300delAT] p.[splice donor variant];[His100Argfs*14]	1	-
c.[109G>A];[235delC] p.[Val37Ile];[Leu79Cysfs*3]	1	-
Total	49 (22.3%)	-
***GJB2* genotypes with a single recessive pathogenic variant**		
c.[-23+1G>A];[wt] p.[splice donor variant];[wt]	5	3
c.[516G>C];[wt] p.[Trp172Cys];[wt]	9	6
c.[-23+1G>A];[79G>A] p.[splice donor variant];[Val27Ile]	1	3
c.[79G>A];[516G>C] p.[Val27Ile];[Trp172Cys]	1	0
c.[235delC];[wt] p.[Leu79Cysfs*3];[wt]	1	0
c.[109G>A];[wt] p.[Val37Ile];[wt]	1	2
c.[79G>A];[109G>A] p.[Val27Ile];[Val37Ile]	0	1
Total	18 (8.2%)	15 (9.6%)
***GJB2* genotypes with benign variants**		
c.[79G>A];[wt] p.[Val27Ile];[wt]	27	31
c.[79G>A;341A>G];[wt] p.[Val27Ile;Glu114Gly];[wt]	15	12
c.[79G>A];[79G>A] p.[Val27Ile];[Val27Ile]	7	6
c.[79G>A];[79G>A;341A>G] p.[Val27Ile];[Val27Ile;Glu114Gly]	1	2
c.[79G>A;341A>G];[571T>C] p.[Val27Ile;Glu114Gly];[Phe191Leu]	1	0
c.[79G>A];[571T>C] p.[Val27Ile];[Phe191Leu]	1	1
c.[457G>A];[wt] p.[Val153Ile];[wt]	1	0
c.[608T>C];[wt] p.[Ile203Thr];[wt]	1	0
c.[79G>A;341A>G];[457G>A] p.[Val27Ile;Glu114Gly];[Val153Ile]	0	1
Total	54 (24.5%)	53 (33.7%)
***GJB2* genotype [wt];[wt]**	99 (45.0%)	89 (56.7%)

* - *GJB2* variations are designated at the nucleotide level (NM_004004.6) and at the amino acid level (NP_003995.2) at the top and bottom of each line, respectively. The *GJB2* sequence variant c.516G>C (p.Trp172Cys) was submitted as ‘possibly pathogenic’ to ClinVar (http://www.ncbi.nlm.nih.gov/clinvar/) and the ClinVar accession number is SCV000852065.

**Table 3:** Frequencies of the *GJB2* gene variations in Tuvinian patients and the control sample.

*GJB2* Gene Sequence Variations	dbSNP ID	Patients (*n* = 184) * Number of Alleles/Frequency	Control Sample (*n* = 157) Number of Alleles/Frequency
***GJB2* pathogenic variants**			
c.516G>C (p.Trp172Cys)	not presented	51/**0.1386**	6/**0.019**
c.-23+1G>A	rs80338940	30/**0.0815**	6/**0.019**
c.235delC	rs80338943	5/**0.0136**	0/**0.0**
c.299_300delAT	rs111033204	2/0.0054	0/0.0
c.109G>A (p.Val37Ile)	rs72474224	2/0.0054	3/0.0096
Total		90/**0.2446**	15/**0.0478**
***GJB2* benign variants**			
c.79G>A (p.Val27Ile)	rs2274084	41/0.1141	50/0.1592
c.[79G>A;341A>G] (p.[Val27Ile;Glu114Gly])	rs2274084 + rs2274083	15/0.0408	15/0.0478
c.571T>C (p.Phe191Leu)	rs397516878	2/0.0054	1/0.0032
c.457G>A (p.Val153Ile)	rs111033186	1/0.0027	1/0.0032
c.608T>C (p.Ile203Thr)	rs76838169	1/0.0027	0/0.0

* - To avoid probable bias due to the presence of related individuals in the total cohort of Tuvinian patients (*n* = 220), only 184 unrelated patients selected by pedigree analysis were used for this allele frequency calculation. Significant (*p* < 0.05) differences in frequencies between group of patients and the control group are shown in bold.

## Supplementary Materials

The following are available online at https://www.mdpi.com/2073-4425/10/6/429/s1, Figure S1: Verification of *cis*-configuration of variants c.79G>A (p.Val27Ile) and c.341A>G (p.Glu114Gly), Figure S2: Additional pedigrees of Tuvinian families demonstrating the segregation of variant c.516G>C (p.Trp172Cys) in homozygous or in compound heterozygous state with HL in affected family members, Figure S3: The familial GJB2 monoallelic cases, Table S1: The detailed data on the patients with a single *GJB2* pathogenic variant.
