# Supplementary material for: Unique Mutational Spectrum of the GJB2 Gene and Its Pathogenic Contribution to Deafness in Tuvinians (Southern Siberia, Russia): A High Prevalence of Rare Variant c.516G>C (p.Trp172Cys)
Source: Genes (Basel). 2019 Jun 5;10(6):429. doi: 10.3390/genes10060429 (PMC6627114; doi:10.3390/genes10060429)

Figure S1. Verification of *cis*-configuration of variants c.79G>A (p.Val27Ile) and c.341A>G (p.Glu114Gly).

A. Verification of *cis*-configuration of variants c.79G>A (p.Val27Ile) and c.341A>G (p.Glu114Gly) by analysis of pedigrees: transmission of allele p.[Val27Ile;Glu114Gly].

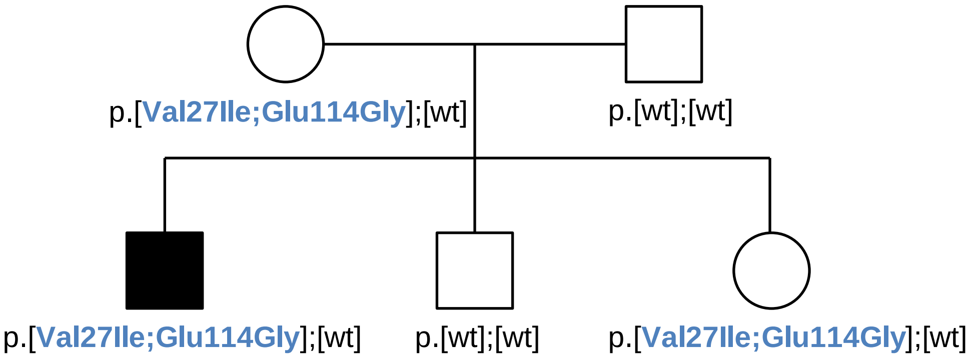

B. An example sequencing trace showing variants c.79G>A (p.Val27Ile) and c.341A>G (p.Glu114Gly) in *cis*-configuration. Conjoint presence of these variants in an examined clone confirmed their *cis*-configuration.

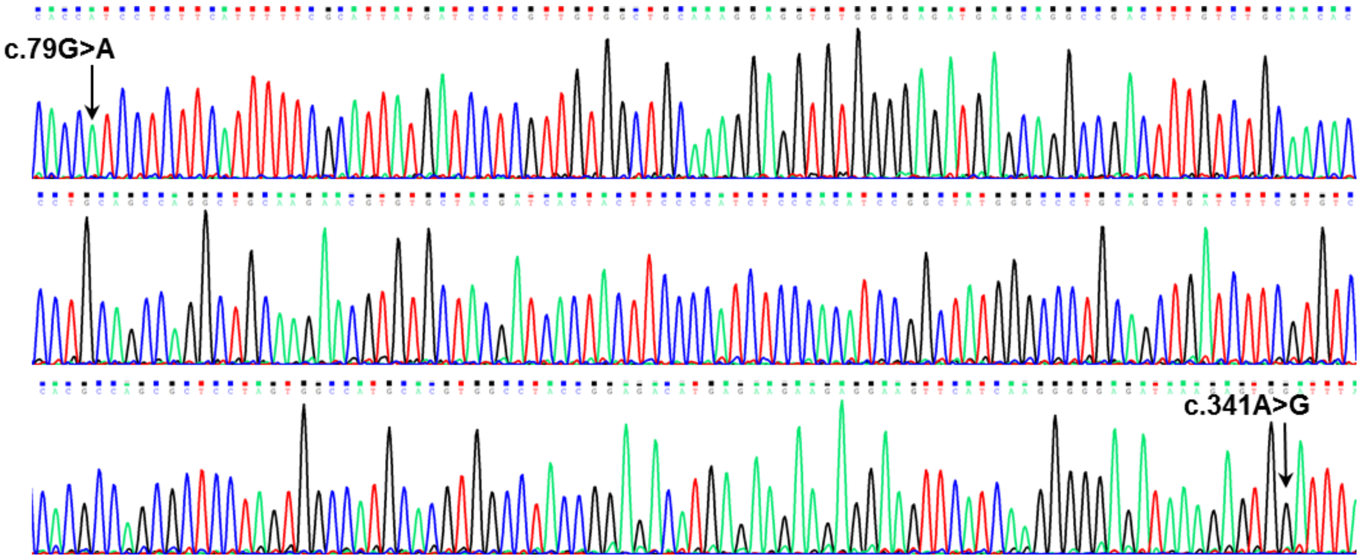

Supplement: Supplementary file 1 [file genes-10-00429-s001.zip › Supplementary materials/Figure S1.pdf]
