# Supplementary material for: Unique Mutational Spectrum of the GJB2 Gene and Its Pathogenic Contribution to Deafness in Tuvinians (Southern Siberia, Russia): A High Prevalence of Rare Variant c.516G>C (p.Trp172Cys)
Source: Genes (Basel). 2019 Jun 5;10(6):429. doi: 10.3390/genes10060429 (PMC6627114; doi:10.3390/genes10060429)

**Figure S2.** Additional pedigrees of Tuvinian families demonstrating the segregation of variant c.516G>C (p.Trp172Cys) in homozygous or in compound heterozygous state with HL in affected family members. Deaf individuals are shown by black symbols. The variant c.516G>C (p.Trp172Cys) is shown by red.

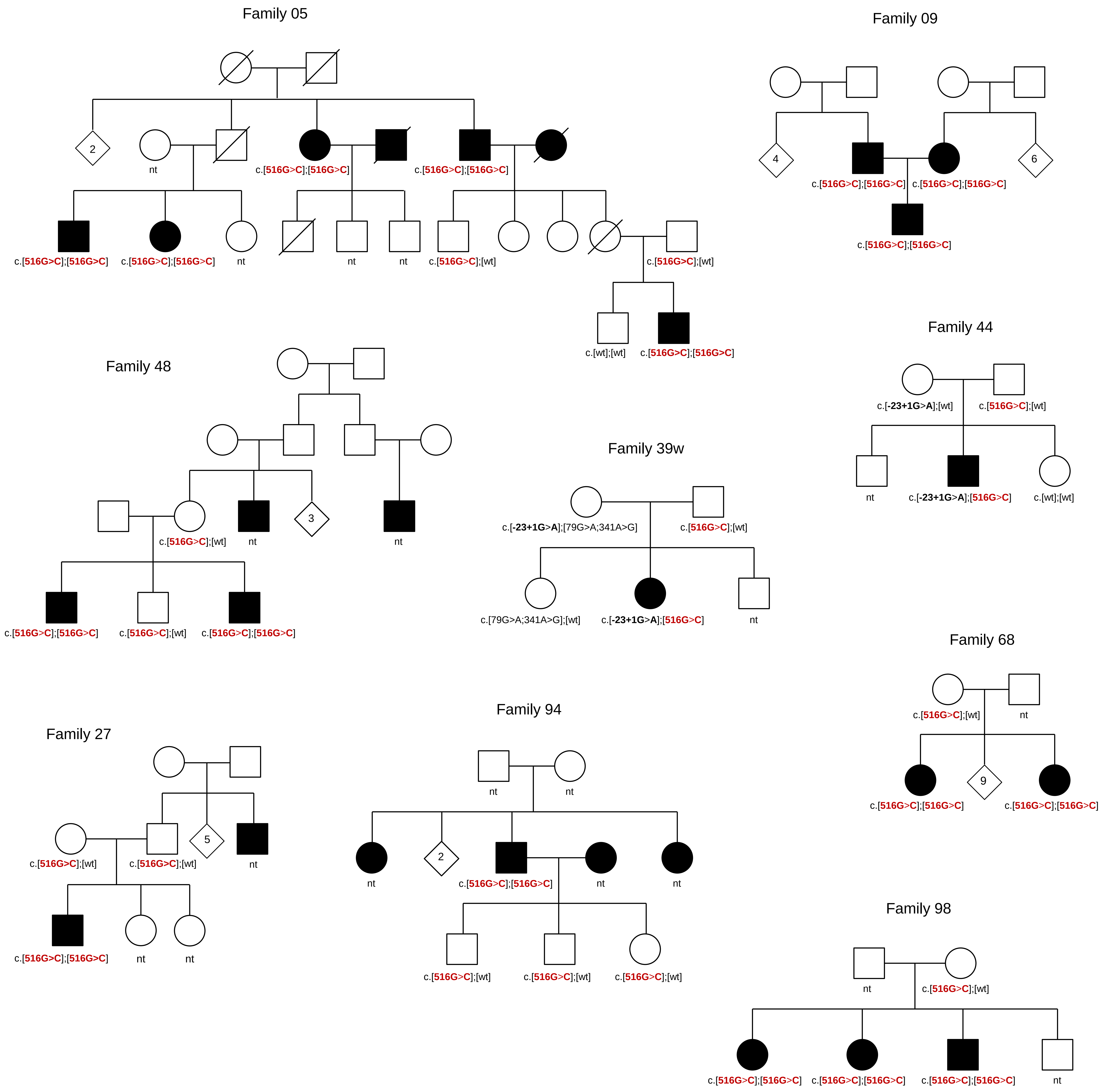

Supplement: Supplementary file 1 [file genes-10-00429-s001.zip › Supplementary materials/Figure S2.pdf]
