# Supplementary material for: Unique Mutational Spectrum of the GJB2 Gene and Its Pathogenic Contribution to Deafness in Tuvinians (Southern Siberia, Russia): A High Prevalence of Rare Variant c.516G>C (p.Trp172Cys)
Source: Genes (Basel). 2019 Jun 5;10(6):429. doi: 10.3390/genes10060429 (PMC6627114; doi:10.3390/genes10060429)

Figure S3. The familial *GJB2* monoallelic cases.

Deaf individuals are shown by black symbols. The patients with *GJB2* monoallelic mutations are shown by blue arrows. The *GJB2* pathogenic variants and the *SLC26A4* pathogenic variants (M1, M2 or M3) are shown by red and green, respectively. Mating types: D x D (including noncomp. D x D), D x N, N x N (*see explanation in text*).

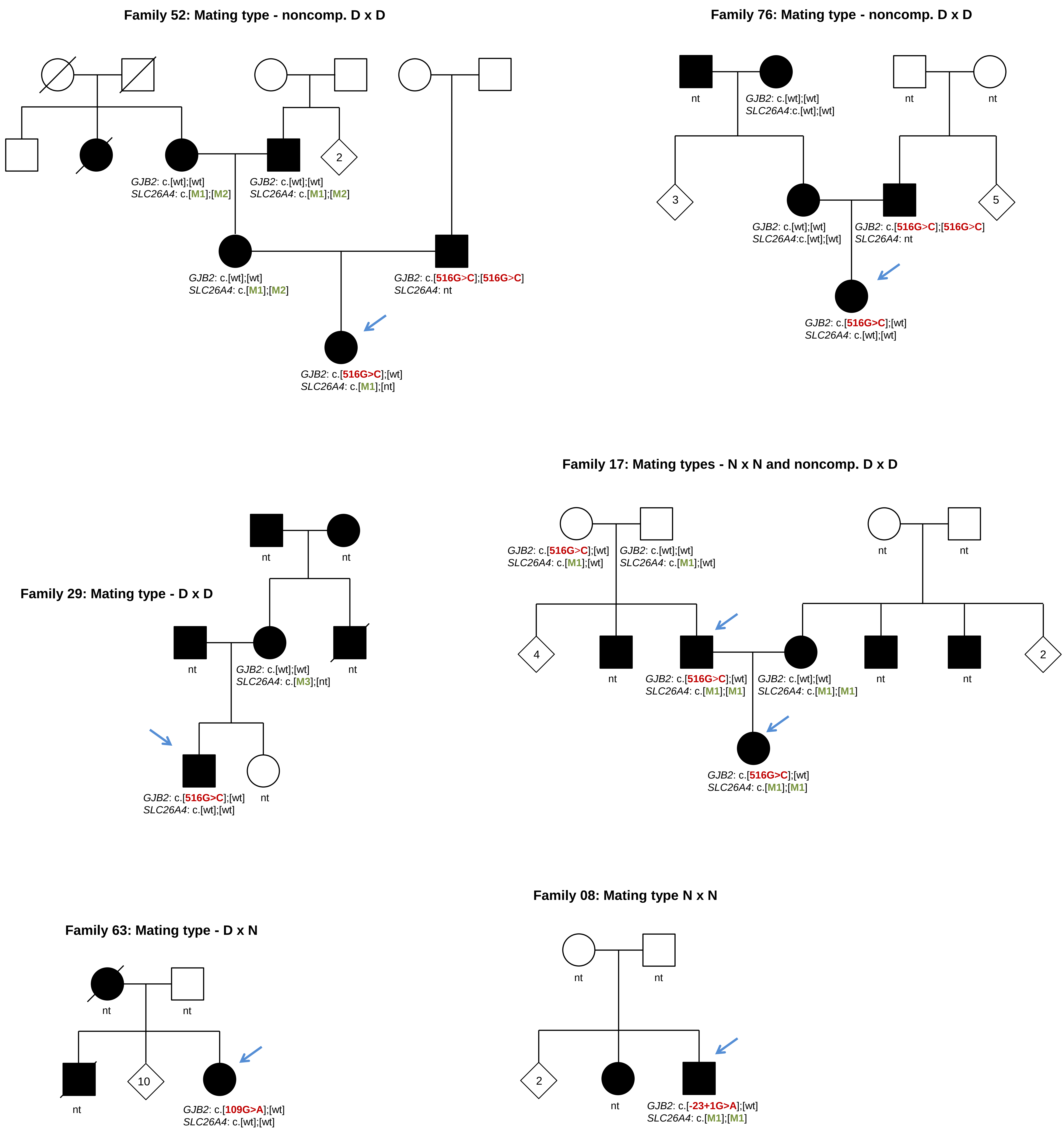

Supplement: Supplementary file 1 [file genes-10-00429-s001.zip › Supplementary materials/Figure S3.pdf]
