# Supplementary material for: Unique Mutational Spectrum of the GJB2 Gene and Its Pathogenic Contribution to Deafness in Tuvinians (Southern Siberia, Russia): A High Prevalence of Rare Variant c.516G>C (p.Trp172Cys)
Source: Genes (Basel). 2019 Jun 5;10(6):429. doi: 10.3390/genes10060429 (PMC6627114; doi:10.3390/genes10060429)
Supplement: Supplementary file 1 [file genes-10-00429-s001.zip › Supplementary materials/Table S1.pdf]

Table S1. The detailed data on the patients with a single *GJB2* pathogenic variant.

	Patients	*GJB2* coding region genotype		Variants in the *GJB2* noncoding regions									Results of the *SLC26A4* testing **	Cases	Family data: mating type of parents and short describtion
			Location	*GJB2* promotor region and noncoding exon 1							*GJB2* splice acceptor site				
			dbSNP	rs9550621	rs117685390	rs9552101	rs1417111166	rs1411911768	rs574815423	rs786204734	rs9578260	rs201895089			
			NM_004004.5:	c.-484T>C	c.-410T>C	c.-369A>G	c.-259C>T	c.-254C>T	c.-216T>G	c.-23G>T	c.-22-12C>T	c.-22-2A>C			
			ClinVar	NA	NA	NA	NA	N/A	Likely benign	Likely pathogenic	Benign/Uncertain-significance	Pathogenic			
			Reference	[Matos et al., 2011]	[Matos et al., 2011]	[Matos et al., 2011]	[Matos et al., 2007]	this study *	[Matos et al., 2011]	[Mani et al., 2009]	[Matos et al., 2011]	[Gandía et al., 2013]			
1	**Tuv_008**	c.[**516G>C**];[wt]		C/C	T/T	G/G	C/C	**C/T**	T/T	G/G	C/C	A/A	no pathogenic *SLC26A4* variants	sporadic	**N x N**: at least one of parents (nt) is potential carrier of *GJB2* pathogenic variant. 1 out of 3 siblings is deaf.
2	**Tuv_011**	c.[**-23+1G>A**];[wt]		C/C	T/T	G/G	C/C	C/C	T/T	G/G	C/C	A/A	**hearing loss due to *SLC26A4***	familial	**N x N:** each of parents (nt) is potential carrier of one *SLC26A4* pathogenic variant and one of them – potential carrier of one *GJB2* pathogenic variant. 2 out of 4 siblings are deaf.
3	**Tuv_033**	c.[**-23+1G>A**];[wt]		C/C	T/T	G/G	C/C	C/C	T/T	G/G	C/C	A/A	no pathogenic *SLC26A4* variants	sporadic	**N x N**: one parent has wild *GJB2* genotype, another - nt (potential carrier of *GJB2* pathogenic variant). 1 out of 4 siblings is deaf.
4	**Tuv_076**	c.[**516G>C**];[wt]		C/C	T/T	G/G	C/C	**C/T**	T/T	G/G	C/C	A/A	**hearing loss due to *SLC26A4***	familial	**noncomp.D x D:** both of parents are homozygotes for *SLC26A4* pathogenic variant, in addition, one of them – carrier of *GJB2* pathogenic variant. The only offspring in this family is deaf.
5	**Tuv_078**	c.[**516G>C**];[wt]		C/C	T/T	G/G	C/C	**C/T**	T/T	G/G	C/C	A/A	single pathogenic *SLC26A4* allele	sporadic	**N x N**: both parents (nt) are potential carriers of *GJB2* or *SLC26A4* pathogenic variants. 1 out of 3 siblings is deaf.
6	**Tuv_080**	c.[**-23+1G>A**];[wt]		C/C	T/T	G/G	C/C	C/C	T/T	G/G	C/C	A/A	no pathogenic *SLC26A4* variants	sporadic	**N x N**: at least one of parents (nt) is potential carrier of *GJB2* pathogenic variant. 1 out of 3 siblings is deaf.
7	**Tuv_093**	c.[**516G>C**];[wt]		C/C	T/T	G/G	C/C	**C/T**	T/T	G/G	C/C	A/A	no pathogenic *SLC26A4* variants	familial	**noncomp.D x D:** one of parents is heterozygote for *SLC26A4* pathogenic variant, another (nt) – potentially carrier of *GJB2* pathogenic variant. The only offspring in this family is deaf.
8	**Tuv_128**	c.[79G>A];[**516G>C**]		C/C	T/C	G/G	C/C	**C/T**	T/T	G/G	C/C	A/A	no pathogenic *SLC26A4* variants	sporadic	**N x N:** at least one of parents (nt) is potential carrier of *GJB2* pathogenic variant. 1 out of 2 siblings is deaf.
9	**Tuv_153**	c.[**-23+1G>A**];[wt]		C/C	T/T	G/G	C/C	C/C	T/T	G/G	C/C	A/A	no pathogenic *SLC26A4* variants	sporadic	**N x N:** at least one of parents (nt) is potential carrier of *GJB2* pathogenic variant. 1 out of 2 siblings is deaf.
10	**Tuv_184**	c.[**-23+1G>A**];[79G>A]		C/C	T/C	G/G	C/C	C/C	T/T	G/G	C/C	A/A	no pathogenic *SLC26A4* variants	sporadic	**N x N**: at least one of parents (nt) is potential carrier of *GJB2* pathogenic variant. 1 out of 3 siblings is deaf.
11	**Tuv_185**	c.[**235delC**];[wt]		C/C	T/T	G/G	C/C	C/C	T/T	G/G	C/C	A/A	no pathogenic *SLC26A4* variants	sporadic	**N x N:** at least one of parents (nt) is potential carrier of *GJB2* pathogenic variant. 1 out of 4 siblings is deaf.
12	**Tuv_190**	c.[**109G>A**];[wt]		C/C	T/C	G/G	C/C	C/C	T/T	G/G	C/C	A/A	no pathogenic *SLC26A4* variants	familial	**D x N:** both parents are not tested, 2 out of 12 siblings are deaf.
13	**Tuv_231**	c.[**516G>C**];[wt]		C/C	T/T	G/G	C/C	**C/T**	T/T	G/G	C/C	A/A	**hearing loss due to *SLC26A4***	sporadic	**N x N:** one of parents is heterozygote for *SLC26A4* pathogenic variant, another – double heterozygote for *GJB2* and *SLC26A4* pathogenic variants. 1 out of 6 siblings is deaf.
14	**Tuv_233**	c.[**-23+1G>A**];[wt]		C/C	T/T	G/G	C/C	C/C	T/T	G/G	C/C	A/A	no pathogenic *SLC26A4* variants	sporadic	**N x N:** at least one of parents (nt) is potential carrier of *GJB2* pathogenic variant. 1 out of 10 siblings is deaf.
15	**Tuv_246**	c.[**516G>C**];[wt]		C/C	T/T	G/G	C/C	**C/T**	T/T	G/G	C/C	A/A	no pathogenic *SLC26A4* variants	sporadic	**N x N:** at least one of parents (nt) is potential carrier of *GJB2* pathogenic variant. 1 out of 10 siblings is deaf.
16	**Tuv_250**	c.[**516G>C**];[wt]		C/C	T/T	G/G	C/C	**C/T**	T/T	G/G	C/C	A/A	no pathogenic *SLC26A4* variants	familial	**noncomp.D x D:** one of parents is homozygote for *GJB2* pathogenic variant, another – wild *GJB2* genotype. The only offspring in this family is deaf.
17	**Tuv_265**	c.[**516G>C**];[wt]		C/C	T/T	G/G	C/C	**C/T**	T/T	G/G	C/C	A/A	**hearing loss due to *SLC26A4***	familial	**N x N:** one of parents is heterozygote for *SLC26A4* pathogenic variant, another – double heterozygote for *GJB2* and *SLC26A4* pathogenic variants. 2 out of 6 siblings are deaf.
18	**Tuv_300**	c.[**516G>C**];[wt]		C/C	T/T	G/G	C/C	**C/T**	T/T	G/G	C/C	A/A	single pathogenic *SLC26A4* allele	familial	**noncomp.D x D:** one of parents is homozygote for *GJB2* pathogenic variant, another – homozygote for *SLC26A4* pathogenic variant. The only offspring in this family is deaf.

* The strong linkage of variant T in rs1411911768 (c.-254C>T) with c.516G>C (p.Trp172Cys) was observed: all heterozygotes for c.516G>C (p.Trp172Cys) have genotype C/T for rs1411911768. This linkage was confirmed also in all examined Tuvinians having c.516G>C (p.Trp172Cys) in homozygous and heterozygous state [Zytsar, personal communication]. ** [Danilchenko, personal communication].

References:

Danilchenko, V.Y. (Institute of Cytology and Genetics, Novosibirsk, Russia). Personal communication, 2019.

Gandía, M.; del Castillo, F.J.; Rodríguez-Álvarez, F.J.; Garrido, G.; Villamar, M.; Calderón, M.; Moreno-Pelayo, M.A.; Moreno, F.; del Castillo, I. A Novel Splice-Site Mutation in the *GJB2* Gene Causing Mild Postlingual Hearing Impairment. *PLoS ONE* **2013**, *8*, e73566.

Mani, R.S.; Ganapathy, A.; Jalvi, R.; Srikumari Srisailapathy, C.R.; Malhotra, V.; Chadha, S.; Agarwal, A.; Ramesh, A.; Rangasayee, R.R.; Anand, A. Functional consequences of novel connexin 26 mutations associated with hereditary hearing loss. *Eur. J. Hum. Genet.* **2009**, *17*, 502–509.

Matos, T.D.; Caria, H.; Simoes-Teixeira, H.; Aasen, T.; Nickel, R.; Jagger, D.J.; O'Neill, A.; Kelsell, D.P.; Fialho, G. A novel hearing loss-related mutation occurring in the *GJB2* basal promoter. *J. Med. Genet.* **2007**, *44*, 721–725.

Matos, T.D.; Simões-Teixeira, H.; Caria, H.; Cascão, R.; Rosa, H.; O'Neill, A.; Dias, Ó.; Andrea, M.E.; Kelsell, D.P.; Fialho, G. Assessing Noncoding Sequence Variants of *GJB2* for Hearing Loss Association. *Genet. Res. Int.* **2011**, *2011*, 1–8.

Zytsar, M.V. (Institute of Cytology and Genetics, Novosibirsk, Russia). Personal communication, 2019.
